# High levels of mortality, exposure to violence and psychological distress experienced by the internally displaced population of Ein Issa camp prior to and during their displacement in Northeast Syria, November 2017

**DOI:** 10.1186/s13031-019-0216-y

**Published:** 2019-07-11

**Authors:** Larissa Vernier, Vanessa Cramond, Maartje Hoetjes, Annick Lenglet, Thomas Hoare, Rami Malaeb, Antonio Isidro Carrion Martin

**Affiliations:** 1 Médecins sans Frontières, Tal Abyad, Syria; 2 Médecins sans Frontières, Plantage Middenlaan 14, 1018 DD Amsterdam, the Netherlands; 3 Médecins sans Frontières, Lower Ground Floor, Chancery Exchange, 10 Furnival Street, London, EC4A 1AB UK; 4 Radboud University Medical Center, Nijmegen, The Netherlands

**Keywords:** Syrian conflict, Internally displaced persons, Morbidity, Mortality, Violence, Psychological distress, Vaccination coverage, Nutrition status

## Abstract

**Background:**

War in Syria has lasted for more than eight years, causing population displacement, collapse of medical and public health services, extensive violence and countless deaths. Since November 2016, military operations in Northeast Syria intensified. In October 2017 a large influx of internally displaced persons (IDPs) arrived to Ein Issa camp, Raqqa governate. Médecins Sans Frontières (MSF) assessed the health status of recently arrived IDPs in Ein Issa camp.

**Methods:**

MSF carried out a cross-sectional survey using simple random sampling between 8 and 18 November 2017, enrolling households who had arrived to Ein Issa camp since 1 October 2017. A questionnaire collected data on demographics, history of displacement, retrospective one-year mortality, two-week morbidities, non-communicable diseases, exposure to violence in the last year and two-week psychological distress symptoms among all household members as well as vaccination status in children aged 6 to 59 months. The latter were also screened for malnutrition. Prevalence estimates and mortality rates were calculated with their 95% confidence interval. Mortality rates were calculated as the number of deaths/10,000 persons/day using the individual person-day contribution of all household members.

**Results:**

MSF surveyed 257 households (1482 participants). They reported 31 deaths in the previous year, resulting in a crude mortality rate of 0.56 deaths/10,000 persons/day (95%CI: 0.39–0.80). Conflict-related violence was the most frequently reported cause of death (64.5%). In the previous year, 31.7% (95%CI: 29.4–34.2) of the participants experienced at least one violent episode. The most frequent type of violence reported was witnessing atrocities (floggings, executions or public body displays); 18.9% (95%CI: 17.0–21.0) of the population and 9.8% (95%CI: 7.9–12.0) of the children under 15 years had witnessed such atrocities. In men over 14 years, 15.8% (95%CI: 11.9–20.8) were detained/kidnapped and 11.3% (95%CI: 8.0–15.8) tortured/beaten/attacked. In the two weeks prior to interview, 14.4% (95%CI: 10.6–19.3) of the respondents felt so hopeless that they did not want to carry on living most of the time.

**Conclusions:**

High levels of mortality, exposure to violence and psychological distress were reported. These survey results increase understanding of the impact of the conflict on the IDP population in Northeast Syria.

## Background

More than half of the population of Syria has been displaced since the war started in 2011 to the end of 2017 [1]. Over five million people have sought refuge in neighboring countries and 6.3 million are currently displaced throughout Syria [1]. Most internally displaced persons (IDPs) inside Syria live in informal settlements and temporary camps with limited security, protection, or access to humanitarian assistance and medical services [2]. The armed conflict, food insecurity, breakdown of government services, limited access to healthcare, insufficient vaccination coverage and shortage of qualified medical staff and supplies have contributed to an excess in morbidity and mortality among civilians living in North Syria since the conflict started [2, 3].

Limited data are available on the humanitarian and health consequences of the conflict within Syria. Between 2011 and 2016, 143,630 conflict-related violent deaths were recorded in non-government-controlled areas, of which 70.6% were civilians [4]. The majority of these civilian conflict-related violent deaths were caused by shelling/airstrikes (57.3%) and adult men were the most affected (71.9%) [4]. In addition to the violence and the directly related mortality, the widespread collapse of medical and public health services in conflict affected areas has caused a reemergence of infectious disease outbreaks and clinical aggravation of non-communicable diseases (NCDs) [2, 5]. Vaccine preventable disease outbreaks have been reported in Syria in the past years, including measles, polio, meningitis and hepatitis A [3, 5–7]. Statistics on national and district level vaccination coverage are lacking, but in a vaccination coverage survey carried out in 2015 in Kobane, Aleppo governate by Médecins Sans Frontières (MSF), only 20.3% of the children under the age of 5 years had received all vaccines due for their age [8]. Reliable prevalence estimates of NCDs among Syrian IDPs are also lacking. However, the prevalence likely reflects the estimates found in Syrian refugees in neighboring countries. In Jordan, 21.8% of Syrian adult refugees reported at least one NCD (including hypertension, diabetes and cardiovascular diseases [CVDs]) [9]. Finally, given the nature of the context, IDPs in North Syria are likely to be experiencing psychological distress. In addition to the lack of access to basic needs (food, clean water, healthcare) [2], violence is experienced and/or witnessed everywhere: in schools, at work, at home and in public spaces such as markets and public streets [10]. Two surveys focusing on mental health and psychosocial needs among Syrian refugees in Jordan, and in Turkey and Lebanon estimated the prevalence of psychological distress at 39 and 42%, respectively [11, 12].

**Fig. 1:**
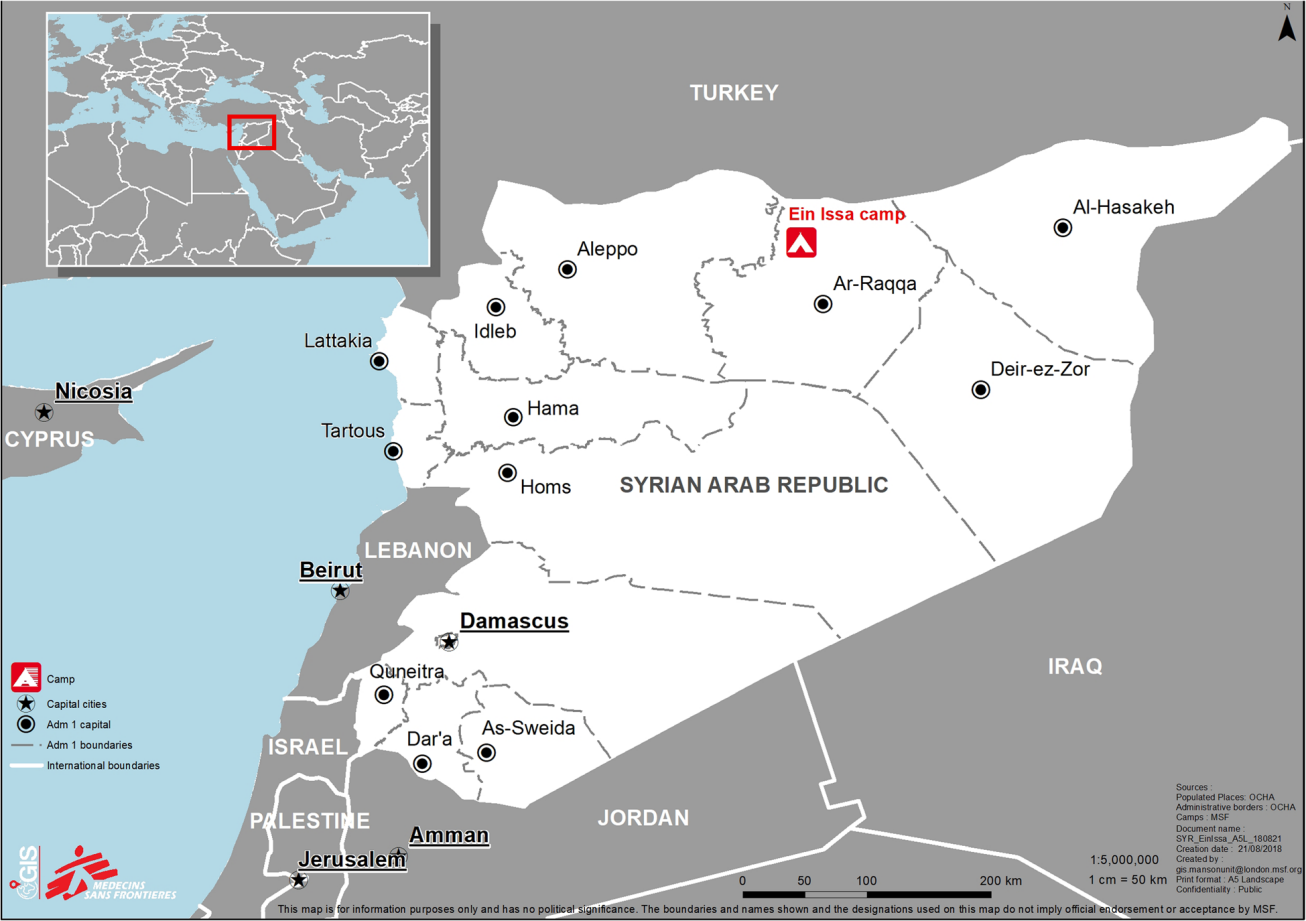
Ein Issa camp, Raqqa governate, Northeast Syria

In November 2016, there was a renewed intensification of the military operations in Northeast Syria. The resulting intensive ground-combat and airstrikes contributed to the deterioration of the population health and living situation triggering considerable population displacements [1]. More than 507,000 people were displaced from and within Deir Ez-Zor and Raqqa governates between November 2016 and November 2017; 146,999 people were displaced in October 2017 alone, during the last weeks of the year-long sieges concentrated in these areas [13]. As a result, a large influx of IDPs arrived to Ein Issa camp, Raqqa governate in October 2017 (Fig. 1). At that time, MSF was supporting one primary healthcare clinic (including mental health support, supplementary feeding and routine immunization activities) and supporting a network of community health workers for disease surveillance purposes.

Given the pervasive violence and insecurity generated by the conflict, international organizations have faced enormous difficulties to be present, to respond to and to bear witness to the humanitarian and medical needs of the IDPs in Northeast Syria, particularly those fleeing from besieged areas. The health indicators and descriptive measurements that inform the health and emergency responses, and provide means to quantify the impact, have so far been largely absent. Considering the large influx of IDPs coming from besieged areas to Ein Issa camp in October 2017, MSF decided to conduct a retrospective mortality and morbidity survey among the recently arrived IDPs in order to orientate health activities, and to increase understanding of the humanitarian impact of the conflict on this population.

## Methods

### Study design and setting

Between 8 and 18 November 2017, MSF conducted a cross-sectional survey among IDPs who had arrived in Ein Issa camp since 1 October 2017. MSF community health workers estimated that the total camp population was composed of 13,840 individuals distributed in 2127 tents (November 2nd 2017).

### Sample size

With 940 total eligible children aged 6–59 months, it required 273 participating children to estimate a 50% vaccination coverage with a simple random sampling design (design effect = 1) and a precision of 5%. Based on an average household size of 5.7 individuals with 20% children aged 6–59 months and accounting for a 10% non-response rate, the number of households to be visited was 275 households.

### Participants

All families living in Ein Issa camp at the time of the survey were considered for inclusion in the survey if they fulfilled the following eligibility criteria: being Syrian internally displaced families and having arrived in the camp since October 1st, 2017. All individuals who had been living under the responsibility of the household head at any time during the last year were considered as household members.

### Definitions

A head of household was defined as any member aged 18 years or older who could give accurate information on the household members, who had lived in the household for most of the last year and who was present at the time of the survey.

Violence was defined following the World Health Organization (WHO) definition i.e. “intentional use of physical force or power, threatened or actual, against oneself, another person, or against a group or community, that either results in or has a high likelihood of resulting in injury, death, psychological harm, maldevelopment, or deprivation” [14].

Conflict-related violence was defined as any episode of violence that was related to a gunshot, landmine/unexploded ordnance or airstrike/shelling.

Disability was defined as any physical disability (i.e. paralysis AND/OR deformation AND/OR amputation leading to mobility impairment), visual impairment/blindness and hearing loss/deafness.

### Sampling

The existing MSF network of community health workers provided a comprehensive camp population and household census including information about demographics, date of arrival, origin as well as location in the camp. After having excluded the non-eligible families, a simple random sampling method was used to select 275 households from this census list. No replacement was done in the case a household was absent or refused to participate in the survey. However, if the selected household was not eligible, or the tent no longer existed, or the tent belonged to one family that owned two tents and had already participated, replacement was done by randomly selecting new households from the census list.

### Data collection

A structured questionnaire was administered to the heads of household during interviews carried out in local Arabic by trained surveyors. All family members present at the time of the interview were able to give inputs. The questionnaire collected data on demographics, history of displacement, retrospective one-year mortality of all household members, two-week recall for morbidities in household members and prevalence of NCDs in household members. MSF also collected information on exposure to violence during the last year and symptoms of psychological distress during the course of the previous two weeks among all household members. For children aged 6–59 months, MSF assessed their vaccination status for polio, measles, BCG and pentavalent (DTPHibHepB) vaccines as reported by caretakers and/or retrieved from the vaccination card.

MSF assessed the mental health status of household members using a standardized assessment tool for mental health developed by the WHO and United Nations High Commission for Refugees (UNHCR) for the Syrian context [15].

All children aged between 6 and 59 months underwent a nutritional status assessment through mid-upper arm circumference (MUAC) measurement and an assessment of bilateral oedema presence. Data were collected on printed forms and entered using EpiData EntryClient version 4.2.0.0 (EpiData Association, Denmark).

### Data analysis

Overall and age/sex-stratified prevalence estimates for the above-mentioned indicators and mortality rates were calculated with their respective 95% confidence interval (95%CI).

Mortality rates were estimated as the number of deaths/10,000 persons/day using the individual person-day contribution of all household members as the denominator. The individual recall period started one year before the interview or on the day of birth and ended on the day before the interview, on the day of death or on the day of last contact for those who left and for which the head of household had no information about their survival.

Overall and vaccine-specific full immunization was calculated for children and was defined as per WHO guidelines for the Syrian Arab Republic [16]: i.e. one dose of BCG vaccine, two doses of measles vaccine, four doses of polio vaccine and four doses of pentavalent vaccine received before the age of two years. We calculated vaccine coverage for 24–59 months for all antigens and for full immunization as all children should have received the above-mentioned antigens at 24 months (except BCG, which is given at birth and was calculated for 6–59 months). Malnutrition thresholds were defined following the MSF guidelines; global acute malnutrition (GAM) was defined as MUAC < 125 mm and/or bilateral oedema and severe acute malnutrition (SAM) as MUAC < 115 mm and/or bilateral oedema [17].

Data analysis was performed using STATA version 14 (StataCorps, College Station, TX, USA).

## Results

### Overall findings

From the 275 randomly selected households, 257 (93.5%) were included in the survey, 8 (2.9%) were absent and 10 (3.6%) refused to participate. Before arriving in Ein Issa camp, 95 (37.0%) of the families used to live in Raqqa governate and 150 (58.4%) in Deir Ez-Zor governate. Most surveyed households (*n* = 187, 72.8%) reported that they had relocated themselves or their family more than once since the beginning of the war in 2011.

**Fig. 2:**
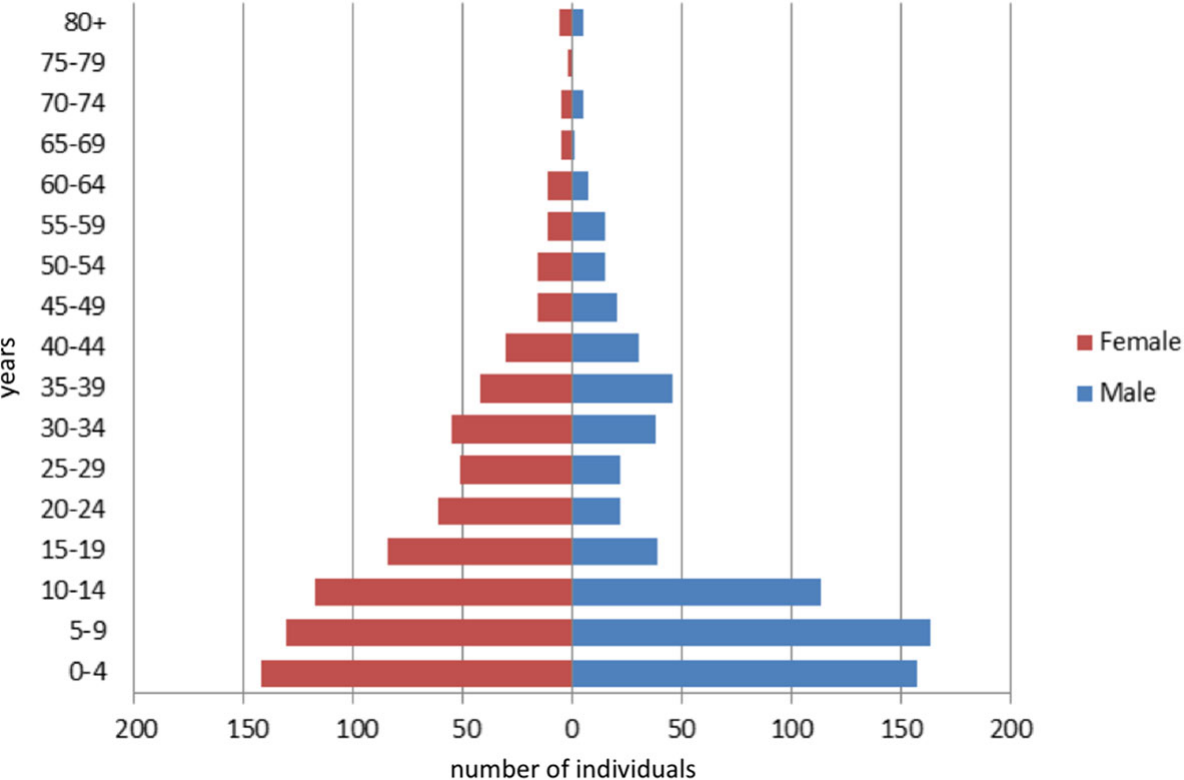
Distribution of participants by sex and age group (*N* = 1482)

A total of 1482 individuals were living in the interviewed households at the time of the survey. The mean household size was 5.8 (standard deviation [SD] = 2.5). The surveyed population was predominantly composed of young people; 20.2% (*n* = 299) were under 5 years old and 55.5% (*n* = 822) were less than 15 years old (Table 1). The median age was 12 years (interquartile range: 6–30 years). The sample comprised 698 (47.1%) men and 784 (52.9%) women, resulting in a male/female-ratio of 0.89. The imbalance was particularly evident in the age group 15–45 years (Fig. 2), in which the male/female-ratio dropped to 0.62. A total of 123 women were pregnant or breastfeeding at the time of the survey, representing 36.8% of women of childbearing age (15–45 years).

**Table 1:** Demographic indicators

Indicator	Total	n (%)
Population under 5 years	1482	299 (20.2%)
Population under 15 years	1482	822 (55.5%)
Men	1482	698 (47.1%)
Women	1482	784 (52.9%)
Pregnant or breastfeeding women	334^a^	123 (36.8%)

^a^ total number of women of childbearing age (15–45 years)

### Retrospective one-year mortality

Between November 2016 and November 2017, 31 deaths were reported among the surveyed population, resulting in a crude mortality rate (CMR) of 0.56/10,000 persons/day (95%CI: 0.39–0.80). Most deaths (*n* = 20, 64.5%) were caused by conflict-related violence. The majority of the deaths (*n* = 26, 83.9%) occurred between June and November 2017, (CMR for this period = 1.04/10,000 persons/day, 95%CI: 0.71–1.53). Almost half of the deaths reported in the year prior to the survey occurred among men aged 15–45 years (*n* = 15, 48.4%), resulting in a specific mortality rate as high as 1.65/10,000 persons/day (95%CI: 1.00–2.74) in this group. Out of the 257 surveyed families, 90 (35.0%) could recall at least one family unit from their extended family or physical neighbors where that entire unit died during the fighting in the previous year.

### Two-week morbidity

The overall prevalence of self-reported illnesses in the previous two weeks was 43.1% (95%CI: 40.6–45.6) (Table 2). Among the reported morbidities, the most frequent were: respiratory complaints (43.9%), gastrointestinal complaints (24.0%), and fever (11.9%). Among women of childbearing age, 5.7% (95%CI: 3.6–8.8) reported gynaecological illnesses in the last two weeks.

**Table 2:** Health-related indicators

Indicator	Total	n	% (95% CI)
Two-week morbidity prevalence (*N* = 1482)			
Any illness	1479*	637	43.1% (40.6–45.6)
Gynaecological illness	334^a^	19	5.7% (3.6–8.8)
Non-communicable disease prevalence among adults (*N* = 582)			
Any NCD	582	165	28.4% (24.8–32.3)
CVD	582	29	5.0% (3.5–7.1)
Hypertension	582	28	4.8% (3.3–6.9)
Disability	582	25	4.3% (2.9–6.3)
Asthma and other chronic respiratory conditions	582	24	4.1% (2.8–6.1)
Musculoskeletal disease (incl. arthritis)	582	23	4.0% (2.6–5.9)
Diabetes	582	22	3.8% (2.5–5.7)
Vaccination coverage in children aged 6–59 months (*N* = 263)			
One dose of BCG	259*	222	85.7% (80.9–89.5)
Four doses of polio vaccine in children aged 24–59 months	172^b^	45	26.2% (20.1–33.3)
Two doses of measles vaccine aged 24–59 months	170^b^	47	27.6% (21.4–34.9)
Four doses of pentavalent vaccine aged 24–59 months	172^b^	35	20.3% (14.9–27.1)
Full immunization for BCG, polio, measles and pentavalent aged 24–59 months	170^b^	24	14.1% (9.6–20.3).
Malnutrition prevalence among children aged 6–59 months (*N* = 263)			
GAM	249*	9	3.6% (1.9–6.8)
SAM	249*	0	–

* Missing values were excluded

^a^ Total number of women of childbearing age (15–45 years)

^b^ Total number of children aged 24–59 months (*N* = 173), missing values excluded

### Non-communicable diseases

Among surveyed adults (≥ 18 years), 28.4% (95%CI: 24.8–32.3) reported to suffer from a NCD (Table 2). The most prevalent NCDs reported were: CVD (5.0%, 95%CI: 3.5–7.1), hypertension (4.8%, 95%CI: 3.3–6.9), disability (4.3, 95%CI: 2.9–6.3), asthma and other chronic respiratory conditions (4.1%, 95%CI: 2.8–6.1), musculoskeletal disease including arthritis (4.0, 95%CI: 2.6–5.9), and diabetes (3.8%, 95%CI: 2.5–5.7).

### Vaccination and nutritional status of children aged 6–59 months

The reported coverage of BCG vaccine among children aged 6–59 months was 85.7% (95%CI: 80.9–89.5) (Table 2). Among children aged 24–59 months, 26.2% (95%CI: 20.1–33.3) had received four doses of polio vaccine, 27.6% (95%CI: 21.4–34.9) had received two doses of measles vaccine, and 20.3% (95%CI: 14.9–27.1) had received four doses of pentavalent. The proportion of children aged 24 to 59 months fully immunized for all investigated antigens was 14.1% (95%CI: 9.6–20.3).

For the malnutrition findings, children with congenital anomalies or birth defects (*n* = 3) were excluded. No severely malnourished children was identified, and the overall GAM prevalence was 3.6% (95%CI: 1.9–6.8).

### Violence exposure in the previous year

In the year preceding the survey, 31.7% (95%CI: 29.4–34.2) of the survey population had been exposed to at least one violent episode (Table 3). The most frequent type of violence reported was a direct witnessing of atrocities such as public floggings, executions or public dead body displays; 18.9% (95%CI: 17.0–21.0) of the population had witnessed such atrocities in their home area. Among children under the age of 15 years, 20.4% (95%CI: 17.8–23.3) had been exposed at least one violent episode and 9.8% (95%CI: 7.9–12.0) had witnessed atrocities. In men aged 15 years and older, 15.8% (95%CI: 11.9–20.8) reported having been detained/kidnapped and 11.3% (95%CI: 8.0–15.8) tortured/beaten/attacked.

**Table 3:** Violence exposure and psychological distress indicators

Indicator	Group	Total	n	% (95%CI)
Violence exposure in the previous year				
Exposed to at least one violent episode	Overall	1478^a^	469	31.7% (29.4–34.2)
Exposed to at least one violent episode	< 15 years	818^a^	167	20.4% (17.8–23.3)
Witnessed atrocities (such as public floggings, executions, public dead body displays)	Overall	1478^a^	279	18.9% (17.0–21.0)
Witnessed atrocities (such as public floggings, executions, public dead body displays)	< 15 years	818^a^	80	9.8% (7.9–12.0)
Detained/kidnapped	Men ≥15 years	265^a^	42	15.8% (11.9–20.8)
Tortured/beaten/attacked	Men ≥15 years	265^a^	30	11.3% (8.0–15.8)
Two-week psychological distress symptoms				
So distressed/disturbed/upset that they were completely or almost completely inactive	> 2 years	1286^a^	485	37.7% (35.1–40.4)
Uninterested in things they used to like most of the time	Respondents	257	75	29.3% (24.0–35.2)
So hopeless that they did not want to carry on living most of the time	Respondents	257	37	14.4% (10.6–19.3)
Bedwetting on at least two occasions	Children aged 5–12 years	446^a^	91	20.4% (16.9–24.4)

^a^ Missing values were excluded

### Two-week psychological distress symptoms

Among participants older than two years of age, 37.7% (95%CI: 35.1–40.4) were so distressed/disturbed/upset in the two weeks prior to the survey that they were completely or almost completely inactive because of these feelings (Table 3). In the same period, 29.3% (95%CI: 24.0–35.2) of the survey respondents were uninterested in things they used to like and 14.4% (95%CI: 10.6–19.3) felt so hopeless that they did not want to carry on living most of the time. Additionally, it was reported that among children aged 5–12 years, 20.4% (95%CI: 16.9–24.4) were bedwetting on at least two occasions in the two weeks prior to the survey.

## Discussion

### Direct effect of the conflict on mortality and demographics

The findings of this survey clearly highlight the direct impact of the violent conflict on the civilian population in Northeast Syria between November 2016 and November 2017. In this period the estimated CMR amongst the IDPs recently arrived in Ein Issa camp was almost five times higher than the Syrian CMR pre-war estimate (according to World Bank data: 0.12/10,000 persons/day [18]). The surveyed population reported that the majority of the deaths were caused by conflict-related violence and occurred between June and November 2017, when the conflict intensified in Northeast Syria with heavy airstrikes and fighting. Conflict and terror was already identified as the first cause of death in Syria in 2016 [19]. This survey showed that men aged 15–45 years carried the brunt of the mortality and this may also explain the deficit of young adult men in the survey population pyramid. It must be noted that the deficit in men of fighting age is typical of conflict settings as they are killed, conscripted to fight, hiding to avoid conscription or migrating to find work [20].

### Mental health burden resulting from the conflict

Most families that were displaced in Ein Issa camp were formerly residing in the besieged areas of Raqqa governate and Deir Ez-zor governate immediately prior to their displacement. The extreme nature of the living conditions in these locations during the siege are well illustrated by the high frequency of violent episodes reported during the survey period, and the extent of that violence (floggings, executions, public body display). Raqqa and Deir Ez-zor governates also suffered from a deterioration of the security situation caused by the intensification of fighting and airstrikes in 2016–2017. The impact of experiencing or witnessing such violent episodes is likely to be reflected in the high reports of psychological trauma from survey participants. In addition to the high exposure to direct and indirect violence, other factors may have added to the psychological burden of those interviewed These factors included the difficult living conditions in the camp, the loss of family members, the constant insecurity and uncertainty about the future, and the relentless displacement demonstrated by this survey (72.8% of the participants had moved more than once since the beginning of the conflict).

The prevalence of psychological distress reported by survey participants was similar to those observed in surveys carried out among Syrian refugees in Jordan, Turkey and Lebanon [11, 12]. This level of distress is noticeably higher than the estimates projected by the WHO for mild or moderate mental disorders in adult populations affected by emergencies (15–20%) [15]. Considerable prevalence of depressive symptoms leading to functional impairment was also found in the survey population. Depression is known to be one of the most prevalent symptoms of emotional distress experienced by populations affected by violence and displacement [21].

A group known to be particularly vulnerable and with psychosocial needs not sufficiently addressed in displaced populations are the children [21]. As shown with this survey, they witnessed or directly experienced war-related violence on a regular basis. The impact of the conflict on children’s mental health might be reflected by the high two-week bedwetting prevalence, a common sign of trauma and distress amongst this age group [22]. Studies implemented in Syrian refugee camps in Lebanon and Turkey confirm the high psychological burden and the high prevalence of post-traumatic stress disorder and depression in children [23].

It is important to highlight that comparison between mental health results from this study with other studies is challenging. Literature on mental health in IDPs is scarce and comparisons with refugees should be made with caution. The IDPs surveyed in the current study have been exposed to a very particular context i.e. populations from urban areas where there has been intense siege and heavy fighting, who now live in an unstable and unsafe setting with limited access to essential services. In addition, reviewed studies use a wide range of measures, distress proxies, and target populations, thus decreasing the pertinence of possible comparison.

### Outbreak potential and vulnerable groups

The Syrian conflict has provided suitable conditions for an increased incidence or re-emergence of communicable diseases through large population movements, high density camp settings, limited access to healthcare, food and water, and low sanitation and hygiene standards. The survey results showed that symptoms potentially indicating communicable diseases represented almost 80% of the reported morbidities. With a vaccination coverage lower than the one found in Kobane, Aleppo governate in 2015 [8], this survey shows that the immunization situation has not improved in Northeast Syria. The antigen-specific vaccination coverages for fully immunized children according to WHO schedule remain well below WHO recommended thresholds i.e. 89% for polio, > 95% for measles and > 79% for most antigens in the pentavalent vaccine [24]. Consequently, population immunity was not met in Ein Issa at the time of the survey, and thus there is an increased risk for outbreaks. Finally, during the course of this survey, MSF identified that amongst the IDPs in Ein Issa, women of childbearing age with gynaecological or pregnancy-related complaints, individuals with NCDs and individuals with a disability restricting their movements were particularly vulnerable in terms of health needs. Despite not rigorously captured by the questionnaire, it has been anecdotally noted that these groups had very limited access to appropriate services or support networks.

### Limitations

Recall bias may have contributed to the underestimation of indicators with a long recall period i.e. violence exposures and vaccination status. Fearing that their reputation and even their safety could be endangered, some participants may also have underreported sensitive information, especially regarding deaths among persons engaged in active fighting and some types of violence exposures (torture, detention and sexual violence). Other indicators such as gynaecological complaints, in women, and psychological distress among men are likely to have been underreported due to cultural and gender norms. On the other hand, some physical and psychological complaints may have been overreported with the expectation of getting more immediate assistance. The wording included in the mental health assessment questionnaire was complex and it is possible that some meaning was lost during local translation.

Furthermore, retrospective mortality surveys are prone to survival bias i.e. deaths in households in which everyone was killed or the few survivors joined another household are not accounted for, resulting in an underestimation of mortality events [20]. Within the Syrian conflict and in particular the 2016–2017 offensives in Northeast Syria, the use of sub-munitions and airstrikes was widespread, causing profound structural damage and collapse of buildings, and thus the death of entire family units simultaneously. In an attempt to understand the potential survival bias in this specific setting, MSF added a question assessing how many participants had lost an entire family unit among their extended family or neighbours. Even though the survival bias was not precisely quantifiable and multiple reporting could not be excluded, this finding indicates that the mortality underestimation is important.

## Conclusions

These confronting results increase understanding of the impact of the conflict on the IDP population in Northeast Syria. The CMR in IDPs in Northeast Syria between November 2016 and 2017 was almost five times higher than pre-war estimates. The majority of these deaths was directly linked to the conflict. The survey demonstrates that people and communities who have fled conflict-affected areas have been highly exposed to violence. The prevalence of distress across all age groups surveyed highlights the immense psychological toll of the conflict on this population, whether due to its violent nature, the relentless forced displacement, the camp living conditions, the constant insecurity and uncertainty about the future, the ongoing forced detention or the multiple losses they have experienced. These results might still reflect the situation of IDPs currently living in camps in Northeast Syria. When implementing in similar context, MSF recommends humanitarian actors to consider the following: prompt mental health and psychosocial support activities, availability and access to quality vaccination services for children under 5 years, and access of vulnerable groups to adequate healthcare such as sexual and reproductive with female health staff, NCDs management activities and widespread outreach activities to reach individuals with restricted movements (as it is best practice).

### Ein Issa camp since November 2017

In the months following this survey, and in accordance with the recommendations provided, MSF and other international and local actors enhanced their presence and activities, including mental health, vaccination and primary health care services. In this period, many families started to return to their places of origin, but many other continued to arrive. At the time of finalizing this article (June 2019) the camp population is almost the same of what was reported in November 2017 (around 13,000 people). Therefore, the demand of humanitarian and health assistance remains high.

## Data Availability

MSF has a managed access system for data sharing. Data are available on request in accordance with MSF’s data sharing policy. Requests for access to data should be made to data.sharing@msf.org.

## Abbreviations

95%CI: 95% confidence interval
CMR: Crude mortality rate
CVD: Cardiovascular disease
GAM: Global acute malnutrition
IDP: Internally displaced person
MSF: Médecins Sans Frontières
MUAC: Mid-upper arm circumference
NCD: Non-communicable disease
SAM: Severe acute malnutrition
UNHCR: United Nations High Commission for Refugees
WHO: World Health Organization
